# Immunization with Single-Cycle SIV Significantly Reduces Viral Loads After an Intravenous Challenge with SIV_mac_239

**DOI:** 10.1371/journal.ppat.1000272

**Published:** 2009-01-23

**Authors:** Bin Jia, Sharon K. Ng, M. Quinn DeGottardi, Michael Piatak, Eloísa Yuste, Angela Carville, Keith G. Mansfield, Wenjun Li, Barbra A. Richardson, Jeffrey D. Lifson, David T. Evans

**Affiliations:** 1 Department of Microbiology and Molecular Genetics, Harvard Medical School, New England Primate Research Center, Southborough, Massachusetts, United States of America; 2 AIDS and Cancer Virus Program, SAIC Frederick, Inc., National Cancer Institute at Frederick, Frederick, Maryland, United States of America; 3 Department of Pathology, Harvard Medical School, New England Primate Research Center, Southborough, Massachusetts, United States of America; 4 Biostatistics Research Group, Division of Preventive and Behavioral Medicine, University of Massachusetts Medical School, Worcester, Massachusetts, United States of America; 5 Department of Biostatistics, University of Washington, Seattle, Washington, United States of America; Oregon National Primate Research Center, United States of America

## Abstract

Strains of simian immunodeficiency virus (SIV) that are limited to a single cycle of infection were evaluated for the ability to elicit protective immunity against wild-type SIV_mac_239 infection of rhesus macaques by two different vaccine regimens. Six animals were inoculated at 8-week intervals with 6 identical doses consisting of a mixture of three different envelope variants of single-cycle SIV (scSIV). Six additional animals were primed with a mixture of cytoplasmic domain-truncated envelope variants of scSIV and boosted with two doses of vesicular stomatitis virus glycoprotein (VSV G) trans-complemented scSIV. While both regimens elicited detectable virus-specific T cell responses, SIV-specific T cell frequencies were more than 10-fold higher after boosting with VSV G trans-complemented scSIV (VSV G scSIV). Broad T cell recognition of multiple viral antigens and Gag-specific CD4^+^ T cell responses were also observed after boosting with VSV G scSIV. With the exception of a single animal in the repeated immunization group, all of the animals became infected following an intravenous challenge with SIV_mac_239. However, significantly lower viral loads and higher memory CD4^+^ T cell counts were observed in both immunized groups relative to an unvaccinated control group. Indeed, both scSIV immunization regimens resulted in containment of SIV_mac_239 replication after challenge that was as good as, if not better than, what has been achieved by other non-persisting vaccine vectors that have been evaluated in this challenge model. Nevertheless, the extent of protection afforded by scSIV was not as good as typically conferred by persistent infection with live, attenuated SIV. These observations have potentially important implications to the design of an effective AIDS vaccine, since they suggest that ongoing stimulation of virus-specific immune responses may be essential to achieving the degree of protection afforded by live, attenuated SIV.

## Author Summary

AIDS vaccine candidates based on recombinant DNA and/or viral vectors stimulate potent cellular immune responses. However, the extent of protection achieved by these vaccines has so far been disappointing. While live, attenuated strains of SIV afford more reliable protection in animal models, there are justifiable safety concerns with the use of live, attenuated HIV-1 in humans. As an experimental vaccine approach designed to uncouple immune activation from ongoing virus replication, we developed a genetic system for producing strains of SIV that are limited to a single cycle of infection. We compared repeated versus prime-boost vaccine regimens with single-cycle SIV for the ability to elicit protective immunity in rhesus macaques against a strain of SIV that is notoriously difficult to control by vaccination. Both vaccine regimens afforded significant containment of virus replication after challenge. Nevertheless, the extent of protection achieved by immunization with single-cycle SIV was not as good as the protection typically provided by persistent infection of animals with live, attenuated SIV. These observations have important implications for the design of an effective AIDS vaccine, since they suggest that ongoing stimulation of virus-specific immune responses may ultimately be necessary for achieving the robust protection afforded by live, attenuated SIV.

## Introduction

The search for a safe and effective AIDS vaccine continues. While live, attenuated strains of SIV afford reliable long-term protection in animal models, at least against closely related challenge viruses, they have the potential to regain a pathogenic phenotype through the accumulation of compensatory genetic changes over prolonged periods of persistent replication *in vivo* [1]–[7]. Hence, there are legitimate safety concerns with the use of live, attenuated HIV-1 as a vaccine approach in people. Vaccine candidates based on recombinant DNA and/or viral vectors are safer and elicit potent cellular immune responses that effectively control virus replication after challenge with the simian-human immunodeficiency virus chimera SHIV89.6P [8]–[11]. However, these vaccines afford only modest protection against SIV challenge strains, such as SIV_mac_239 and SIV_mac_251, that express neutralization-resistant, CCR5-tropic envelope glycoproteins typical of most primary HIV-1 field isolates [12]–[17].

The predictive validity of the more rigorous SIV challenge model as an indicator of vaccine efficacy in humans was recently supported by the failure of a replication-defective, recombinant adenovirus type 5 (rAd5) vaccine candidate to protect against HIV-1 infection in a high profile clinical trial [18]–[23]. In a phase IIb proof-of-concept trial, nearly 3000 participants were immunized at 0, 1 and 6 months with rAd5 vectors expressing HIV-1 clade B *gag*, *pol* and *nef* genes, or a placebo control [18],[19]. The trial was halted after the data safety monitoring board, at its first interim analysis, determined that the vaccine not only failed to prevent infection, but failed to reduce viral loads in immunized individuals who later became infected [18],[19]. These disappointing results have further diminished optimism that similar vaccine approaches might provide better protection in future trials [18],[19],[24]. Thus, there is an urgent need to continue to pursue innovative vaccine concepts that may afford more promising safety and efficacy profiles.

It is presently unclear whether persistent, low-level virus replication, and associated stimulation of virus-specific immune responses, is a prerequisite for the robust protection afforded by infection of animals with live, attenuated strains of SIV. As an experimental AIDS vaccine approach designed to uncouple immune activation from ongoing virus replication and turnover of CD4^+^ lymphocytes, we and others have developed genetic systems for producing strains of SIV that are limited to a single cycle of infection [25]–[27]. Our approach is based on a two-plasmid system for producing Gag-Pol-complemented SIV with mutations in a *cis*-acting sequence required for ribosomal frameshifting between the *gag* and *pol* reading frames [25],[26]. One plasmid carries a full-length proviral genome with mutations in the *gag-pol*-frameshift site to prevent Pol translation, and a second plasmid carries a Gag-Pol-expression construct that also has mutations in the frameshift site [25],[26]. Co-transfection of both plasmids into the same cells results in the release of Gag-Pol-complemented virus particles that package a *pol*-deficient viral genome. Cells infected with this virus express all of the viral gene products except Pol and release immature virions that cannot complete subsequent rounds of infection [25],[26]. Since neither construct retains a functional frameshift site, high-titer stocks of single-cycle SIV (scSIV) can be produced without generating replication-competent virus through recombination [25],[26].

In preliminary studies, viral RNA loads reflecting the production of non-infectious progeny virus by scSIV-infected cells were measurable in plasma after inoculation of macaques with concentrated doses of scSIV [27]–[29]. Progressive reductions in these transient peaks of viremia were observed after three successive doses, and the rank order of peak viral loads after the last dose of scSIV was the same as the rank order of peak viral loads after challenge [28]. These observations suggested that protective immunity might improve with repeated immunization and that the ability to contain scSIV infection after repeated immunizations might ultimately be predictive of the ability to contain wild-type SIV infection after challenge; a hypothesis we have termed “immunization to extinction.”

Protective immunity may also be improved by maximizing the stimulation of virus-specific T cell responses. While a number of factors can influence the development of T cell memory, results from murine systems indicate that the size of the memory T cell population is ultimately determined by the number of activated T cells driven to proliferate during the process of clonal expansion [30],[31]. All other factors being equal, the magnitude of antigen presentation largely determines the extent of T cell activation. Thus, we reasoned that approaches designed to maximize the infectivity of scSIV, and the frequency of scSIV-infected antigen presenting cells in immunized animals, should increase both the size and longevity of the memory T cell population.

In the present study, we compared two different immunization regimens with scSIV for the ability to contain virus replication after an intravenous challenge with SIV_mac_239. One group of animals was inoculated with six identical doses of the same cryopreserved stocks of scSIV to determine if repeated immunization would promote the maturation of virus-specific immune responses. A second group of animals was primed with scSIV strains expressing envelope glycoproteins with truncated cytoplasmic tails and boosted with VSV G trans-complemented scSIV to maximize infection, antigen presentation and the stimulation of virus-specific T cell responses. Despite differences in the magnitude of virus-specific T cell responses elicited, both immunization regimens resulted in statistically significant reductions in viral loads and better preservation of memory CD4^+^ T cell subsets after challenge compared to unimmunized control animals.

## Results

### Repeated versus prime and boost immunizaton with single-cycle SIV

Rhesus macaques were immunized with single-cycle SIV by two different vaccine regimens to compare the effects of frequency of inoculation versus infectious dose of the inoculum on the development of protective immunity. One group of animals (Group A) was repeatedly immunized with the same dose of scSIV to determine if mimicking persistent infection through repeated antigenic stimulation would lead to the progressive maturation of virus-specific immune responses and incremental reductions in single-cycle viral loads predictive of the ability to contain wild-type SIV replication after challenge. A second group of animals (Group B) was immunized by a prime and boost regimen designed to maximize scSIV infection, antigen presentation and the stimulation of virus-specific T cell responses to determine if higher overall T cell responses might result in better control of SIV infection after challenge.

Three different envelope variants of single-cycle SIV expressing full-length (TM_open_) or truncated (TM_stop_) forms of the 239, 316 and 155T3 envelope glycoproteins were used for immunization [32]. The 239 envelope uses CCR5 as a co-receptor for infection of predominantly memory CD4^+^ T cells [32],[33]. The 316 envelope also uses CCR5, but differs from the 239 envelope by 6 amino acids in gp120 which result in a 50- to 100-fold enhancement in infectivity for primary macrophages in culture [25],[32],[34]. The 155T3 envelope, which differs from the 239 envelope by 22 amino acids in gp120, uses CXCR4 rather than CCR5 as a co-receptor for infection of both naive and memory CD4^+^ T lymphocytes [32],[33]. These three envelopes were selected to ensure infection of a diverse population of antigen presenting cells by scSIV and to potentially broaden envelope-specific antibody responses. For each of these envelope variants, TM_stop_ strains of scSIV were created by introducing a glutamic acid to stop-codon change at position 767 (E767*) in the gp41 cytoplasmic tail. The E767* mutation was present in the original isolate of SIV_mac_316 and may represent a naturally selected change to facilitate virus replication in macrophage [34]. Truncation of the gp41 cytoplasmic tail at this position was also shown to increase envelope glycoprotein incorporation into virions and to enhance virus infectivity [32],[35],[36]. To facilitate the stimulation of virus-specific CD8^+^ T cell responses, mutations in *nef* that eliminate residues required for MHC class I downregulation were also included in each strain [28],[37],[38].

**Figure 1:**
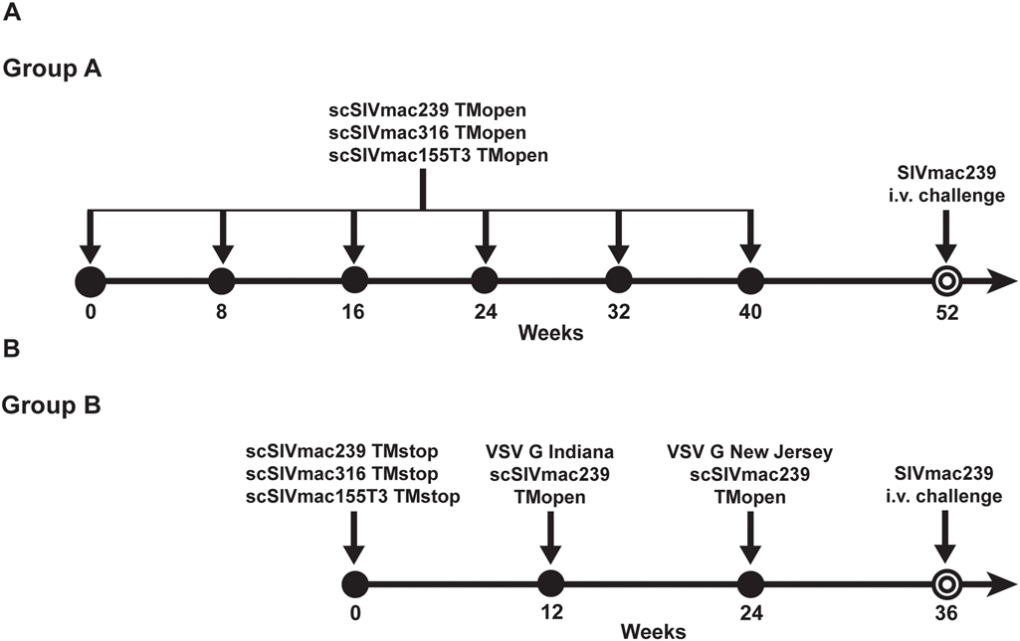
Rhesus macaques were immunized with single-cycle SIV by two different regimens. (A) Six animals in Group A were inoculated with 6 identical doses of scSIV at 8-week intervals. Each dose consisted of a mixture of scSIV_mac_239 TM_open_, scSIV_mac_316 TM_open_, and scSIV_mac_155T3 TM_open_ (5 µg p27 eq. each). (B) Six animals in Group B were primed with a mixture of scSIV_mac_239 TM_stop_, scSIV_mac_316 TM_stop_, and scSIV_mac_155T3 TM_stop_ (5 µg p27 eq. each) and boosted on weeks 12 and 24 with VSV G trans-complemented scSIV (10 and 13 µg p27 eq., respectively). Single-cycle SIV_mac_239 TM_open_ was trans-complemented with the Indiana serotype of VSV G for the first boost (week 12) and the New Jersey serotype of VSV G for the second boost (week 24). (A,B) Twelve weeks after the last dose of scSIV, Groups A and B and four unvaccinated control animals were challenged intravenously (i.v.) with SIV_mac_239.

In Group A, six animals were immunized intravenously at 8-week intervals with 6 identical doses of the same cryopreserved stocks of scSIV. Each dose contained a mixture of three scSIV strains expressing full-length envelope glycoproteins, scSIV_mac_239 TM_open_, scSIV_mac_316 TM_open_ and scSIV_mac_155T3 TM_open_ (Figure 1A) [32]. In Group B, six additional animals were primed intravenously with a mixture of the envelope cytoplasmic tail-truncated strains, scSIV_mac_239 TM_stop_, scSIV_mac_316 TM_stop_ and scSIV_mac_155T3 TM_stop_. The animals in Group B were then boosted intravenously with scSIV trans-complemented with the vesicular stomatitis virus glycoprotein (VSV G) on weeks 12 and 24 (Figure 1B). Although it would have been possible to trans-complement any of the three envelope variants of scSIV with VSV G, only scSIV_mac_239 TM_open_ was used for these booster inoculations. The selection of scSIV_mac_239 TM_open_ was based on an effort to focus strain-specific antibody responses on the envelope glycoprotein of SIV_mac_239. However, to prevent neutralization of the second boost by VSV G-specific antibodies, two different serotypes of VSV G were used for each inoculation [39]. The first boost was trans-complemented with the Indiana serotype of VSV G (VSV G_I_ scSIV) and the second boost was trans-complemented with the New Jersey serotype of VSV G (VSV G_NJ_ scSIV).

### Plasma viral RNA loads were detectable after each dose of single-cycle SIV

**Figure 2:**
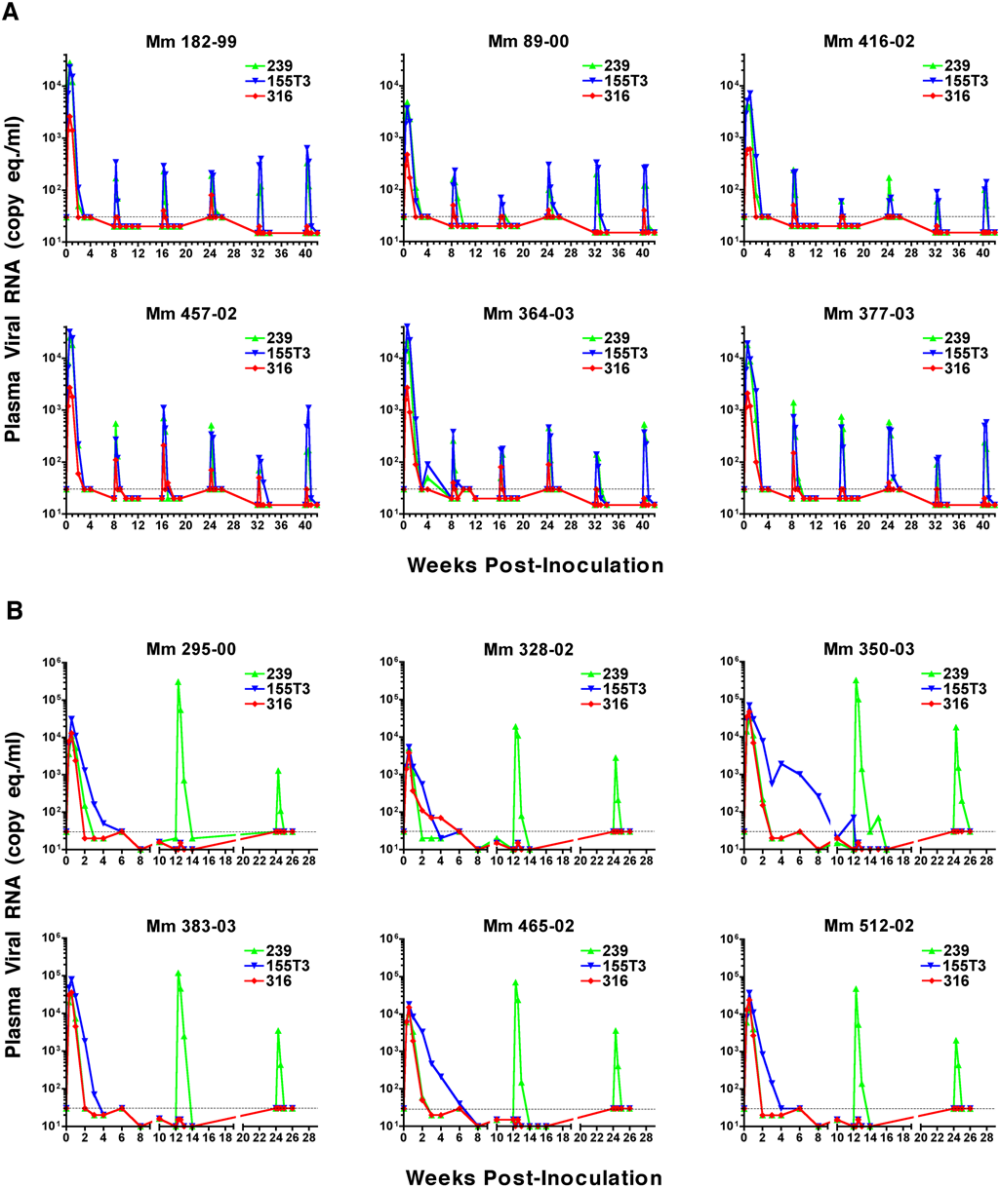
Viral RNA loads were detectable in plasma after each dose of single-cycle SIV. (A) Animals in Group A were inoculated with 6 identical doses of the same cryopreserved stocks of scSIV_mac_239 TM_open_, scSIV_mac_316 TM_open_, and scSIV_mac_155T3 TM_open_ at 8-week intervals. (B) Animals in Group B were primed with a mixture of scSIV_mac_239 TM_stop_, scSIV_mac_316 TM_stop_, and scSIV_mac_155T3 TM_stop_ and boosted with VSV G trans-complemented scSIV_mac_239 TM_open_ on weeks 12 and 24. Viral loads were measured independently for each strain of scSIV using a quantitative multiplex real-time RT-PCR assay specific for unique sequence tags (*ggr*, *cao*, and *gsa*) engineered into each viral genome [32]. The threshold of detection for this assay was 30 RNA copy eq./ml (dotted line).

Plasma viral RNA loads were measured independently for each envelope variant of scSIV using a quantitative, multiplex, real-time RT-PCR assay specific for unique sequence tags cloned into the *pol*-locus of each strain [32]. Measurable levels of virion-associated viral RNA were transiently detectable in plasma after each dose (Figure 2). Since SIV particles are rapidly cleared from plasma with an estimated half-life of a few minutes [40]–[42], these viral load measurements reflect the ongoing release of non-infectious particles from scSIV-infected cells.

In Group A, viral loads peaked on day 4 after the first inoculation and declined below the limit of detection (<30 RNA copy eq./ml) within 3 to 4 weeks (Figure 2A). Geometric mean peak viral loads after the first dose were 1.34×10^4^, 1.49×10^3^ and 1.48×10^4^ RNA copy eq./ml for scSIV_mac_239, scSIV_mac_316 and scSIV_mac_155T3 respectively. After the second dose, peak viral loads occurred on day 2 and were 40-fold lower for scSIV_mac_239 (P = 0.001), 25-fold lower for scSIV_mac_316 (P<0.001) and 50-fold lower for scSIV_mac_155T3 (P<0.001) (Figure 2A). Since the inocula were identical for each dose (same volume of the same cryopreserved stocks), the lower peak and more rapid clearance of viremia after the second inoculation almost certainly reflects the elimination of scSIV-infected cells by virus-specific immune responses. Further reductions in peak viremia were also observed after the fifth and sixth inoculations for scSIV_mac_316 (P = 0.008 and P = 0.02), but not for scSIV_mac_239 or scSIV_mac_155T3 (P>0.05 for all comparisons) (Figure 2A). Since SIV_mac_316 is considerably more sensitive to neutralizing antibodies than SIV_mac_239 or SIV_mac_155T3, better containment of scSIV_mac_316 may reflect the affinity maturation of envelope-specific neutralizing antibodies [43],[44]. However, this was not supported by the results of neutralization assays, since only one of the six animals in this group had detectable neutralizing antibody titers to SIV_mac_316 at the time of challenge. Therefore, other factors, perhaps related to the lower infectivity of scSIV_mac_316, may have contributed to the better containment of viremia for this strain.

In Group B, viral loads also peaked on day 4 after the first inoculation, and with the exception of scSIV_mac_155T3 TM_stop_, were resolved below the limit of detection within 3 to 6 weeks. Consistent with the infectivity enhancement associated with truncation of the gp41 cytoplasmic tail [35],[36], peak viral loads were 4.1-, 6.6- and 56-fold higher after the first dose for TM_stop_ versus TM_open_ scSIV_mac_239, scSIV_mac_155T3 and scSIV_mac_316 [32]. A delay in the clearance of viremia was also observed for scSIV_mac_155T3 TM_stop_, particularly in animals Mm 350-03 and Mm 465-02 (Figure 2B). The more prolonged period of viremia for scSIV_mac_155T3 TM_stop_ was significant based on area under the curve comparisons with scSIV_mac_239 TM_stop_ and scSIV_mac_316 TM_stop_ [32], and may reflect preferential infection of a CD4^+^CXCR4^+^ target cell population that is less susceptible to the cytopathic effects of infection or more resistant to clearance by virus-specific immune responses.

Trans-complementation of scSIV with VSV G resulted in a dramatic infectivity enhancement, presumably by enabling CD4− and chemokine receptor-independent entry of scSIV into cell types that are not normally permissive for SIV infection (Figure 2B). Two days after boosting with VSV G_I_ scSIV, peak viremia was 1.37×10^5^ RNA copy eq./ml. Relative to the first two doses of non-trans-complemented scSIV_mac_239 TM_open_ in Group A, this represents a 10-fold increase over the first peak (P = 0.007) and a 415-fold increase over the second peak (P<0.001). The infectivity enhancement for VSV G_NJ_ scSIV was less dramatic, but was still 4-fold higher than the second non-transcomplemented dose of scSIV_mac_239 TM_open_ in Group A (P = 0.03).

### Repeated immunization with single-cycle SIV did not result in progressive increases in virus-specific T cell frequencies

**Figure 3:**
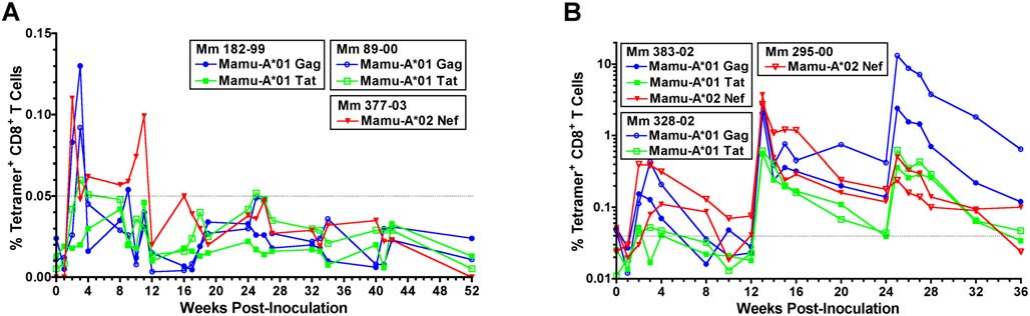
Boosting with VSV G trans-complemented single-cycle SIV significantly increases virus-specific CD8^+^ T cell frequencies. Responses are shown for Mm 182-99, Mm 89-00, and Mm 377-03 in Group A (A), and Mm 383-02, Mm 328-02, and Mm 295-00 in Group B (B). Whole blood was stained with monoclonal antibodies to CD3 and CD8, and one of the following MHC class I tetramers; Mamu-A*01-Gag_181–189_, Mamu-A*01-Tat_28–35_, or Mamu-A*02-Nef_159–167_. Samples were analyzed by flow cytometry, and the percentage of tetramer-positive cells was determined at each time point after gating on the CD3^+^CD8^+^ lymphocyte population. Responses greater than 0.05% (dashed line) are considered positive.

**Figure 4:**
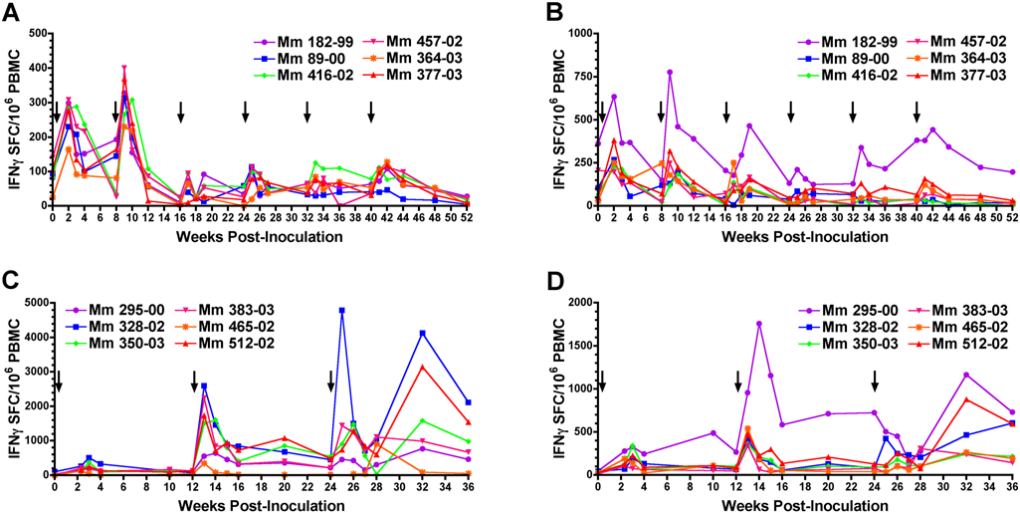
SIV Gag- and Env-specific IFNγ T cell responses were boosted by VSV G trans-complemented single-cycle SIV. Gag- and Env-specific IFNγ T cell responses are shown for Group A (A,B) and Group B (C,D), respectively. PBMCs were stimulated with pools of overlapping peptides representing the complete amino acid sequences of the SIV Gag and Env proteins, and IFNγ-producing cells were enumerated after an overnight incubation in ELISPOT assays. The average number of spot-forming cells (SFC) per million PBMC and the standard deviation (error bars) were determined from duplicate wells set at 3×10^5^ or 1×10^5^ cells per well.

SIV-specific CD8^+^ T cell responses to immunodominant epitopes in Gag, Tat and Nef were monitored in Mamu-A*01 and -A*02 positive animals by directly staining peripheral blood with MHC Class I tetramers. CD8^+^ T cell responses were detectable in Group A animals after the first two inoculations. However, subsequent rounds of inoculation failed to boost CD8^+^ T cell frequencies above the threshold of detection (Figure 3A). Longitudinal analysis of virus-specific T cell responses by IFNγ ELISPOT assays revealed a similar pattern. Primary responses in Group A peaked within 2 to 3 weeks after the first inoculation ranging from 165 to 308 (mean 261) spot-forming cells (SFC) per million PBMC to Gag and from 198 to 635 (mean 327) SFC per million PBMC to Env (Figure 4A and 4B). While recall responses of comparable magnitude were observed after the second inoculation, subsequent rounds of re-inoculation did not result in additional increases in these virus-specific T cell frequencies (Figure 4A and 4B). Thus, despite detectable plasma viremia confirming the take of infection with each dose of scSIV, repeated inoculation did not drive additional expansion of virus-specific T cell responses.

### Boosting with VSV G trans-complemented scSIV increased virus-specific T cell frequencies and resulted in broad T cell recognition of multiple viral antigens

Primary SIV-specific CD8^+^ T cell responses in Group B were similar to primary responses in Group A. However, these responses increased dramatically after boosting with VSV G trans-complemented scSIV. One week after the first boost, the percentages of SIV-specific CD8^+^ T cells in peripheral blood increased 7- to 33-fold for Mamu-A*02 Nef_159–167_ (2.8% to 3.7% CD8^+^ T cells), 7- to 13-fold for Mamu-A*01 Gag_181–189_ (2.0% to 2.8% CD8^+^ T cells) and 12- to 14-fold for Mamu-A*01-Tat_28–35_ (0.55% to 0.61% CD8^+^ T cells) (Figure 3B). Additional recall responses were observed after the second boost. However, with the exception of one animal that made a Mamu-A*01 Gag_181–189_-specific response that exceeded 13% of the circulating CD8^+^ T cell population (Figure 3B), these responses were generally lower reflecting the lower take of infection for VSV G_NJ_ scSIV than for VSV G_I_ scSIV (Figure 2B). Nevertheless, the majority of these CD8^+^ T cell responses remained above the threshold of detection until the time of challenge twelve weeks later, indicating the establishment of a memory CD8^+^ T cell population.

Longitudinal analysis of IFNγ T cell responses in Group B also demonstrated significant expansion of virus-specific T cell responses after boosting with VSV G trans-complemented scSIV. Peak primary responses ranged from 122 to 506 (mean 273) SFC per million PBMC to Gag and from 105 to 341 (mean 230) SFC per million PBMC to Env (Figure 4C and 4D). One week after the first boost, Gag-specific responses were 5.5-fold higher (mean 1496, range 350 to 2595 SFC per million PBMC, P<0.001) and Env-specific responses were 2.3-fold higher (mean 518, range 345 to 957 SFC per million PBMC, P = 0.007) than peak primary responses. Additional recall responses were observed after the second boost (Figure 4C and 4D), and consistent with MHC class I tetramer staining, the animal with the highest IFNγ T cell response to Gag was the same animal with the unusually high frequency of Mamu-A*01 Gag_181–189_-specific CD8^+^ T cells at week 25.

**Figure 5:**
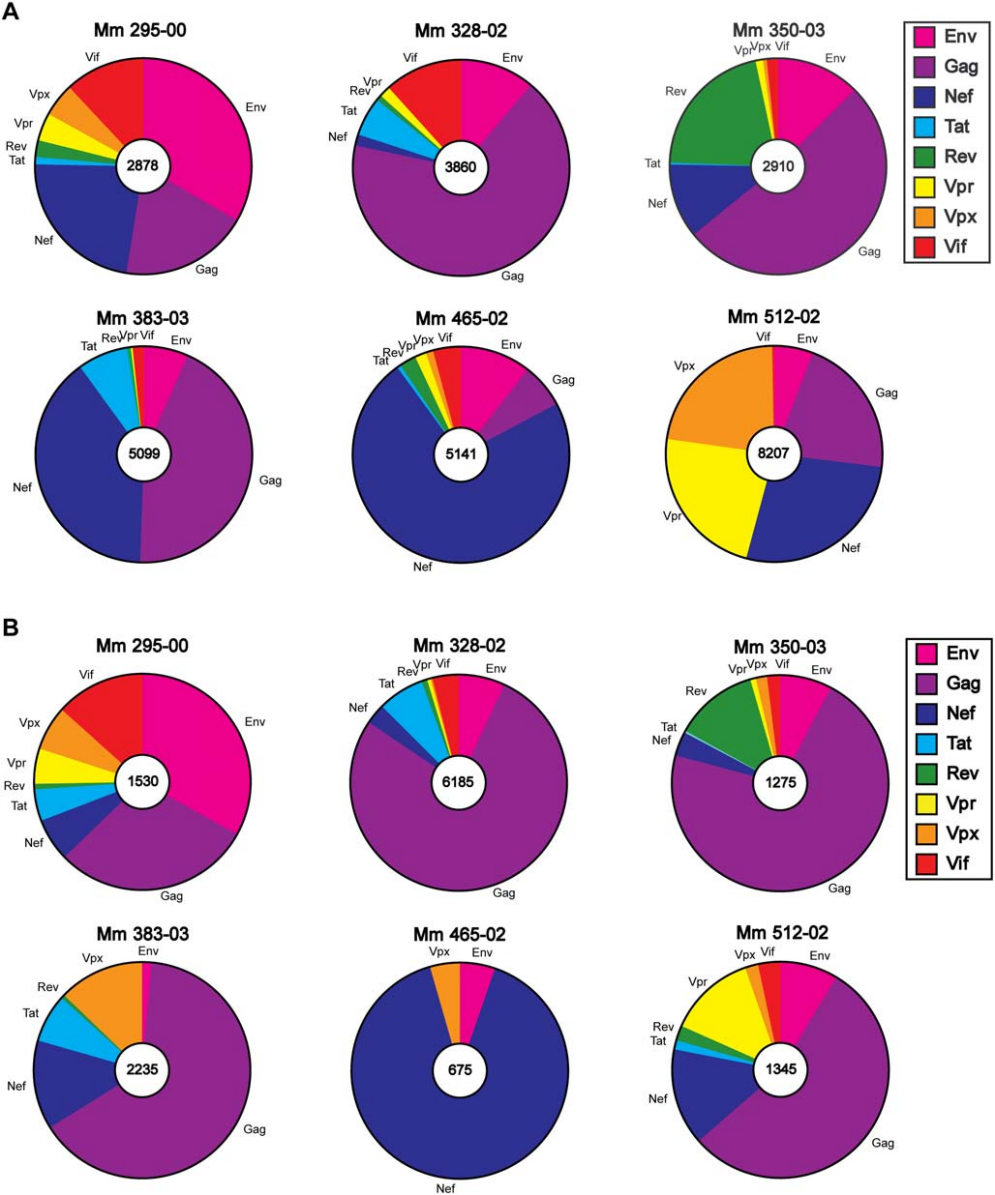
Broad T cell recognition of each of the eight viral gene products expressed by single-cycle SIV. IFNγ ELISPOT responses are shown for Group B animals one week after each boost with VSV G trans-complemented scSIV, week 13 (A) and week 25 (B). Total responses to all 8 viral proteins are indicated in the center of each plot in SFC per million PBMC.

Whole-proteome IFNγ ELISPOT assays also revealed broad T cell recognition of each of the viral gene products expressed by scSIV. The distribution of IFNγ T cell responses to each viral antigen for the animals in Group B is illustrated at week 13 (Figure 5A) and at week 25 (Figure 5B). Although there was considerable animal-to-animal variation in the pattern of responses reflecting the MHC diversity of these outbred animals, the distribution of these responses was relatively stable after each boost. Dominant T cell responses were directed against Gag in three animals (Mm 328-02, Mm 350-03 and Mm 383-03), Env in one animal (Mm 295-00) and Nef in another (Mm 465-02). Only one animal (Mm 512-02) exhibited a notable shift from a diverse, and relatively even distribution of responses to Gag, Nef, Vpr and Vpx after the first boost, to a predominantly Gag-specific response after the second boost (Figure 5). Hence, these results confirm the activation of broad T cell responses capable of targeting each of the 8 viral gene products expressed by scSIV.

### Immunization with single-cycle SIV elicits virus-specific CD4^+^ T cell responses

**Figure 6:**
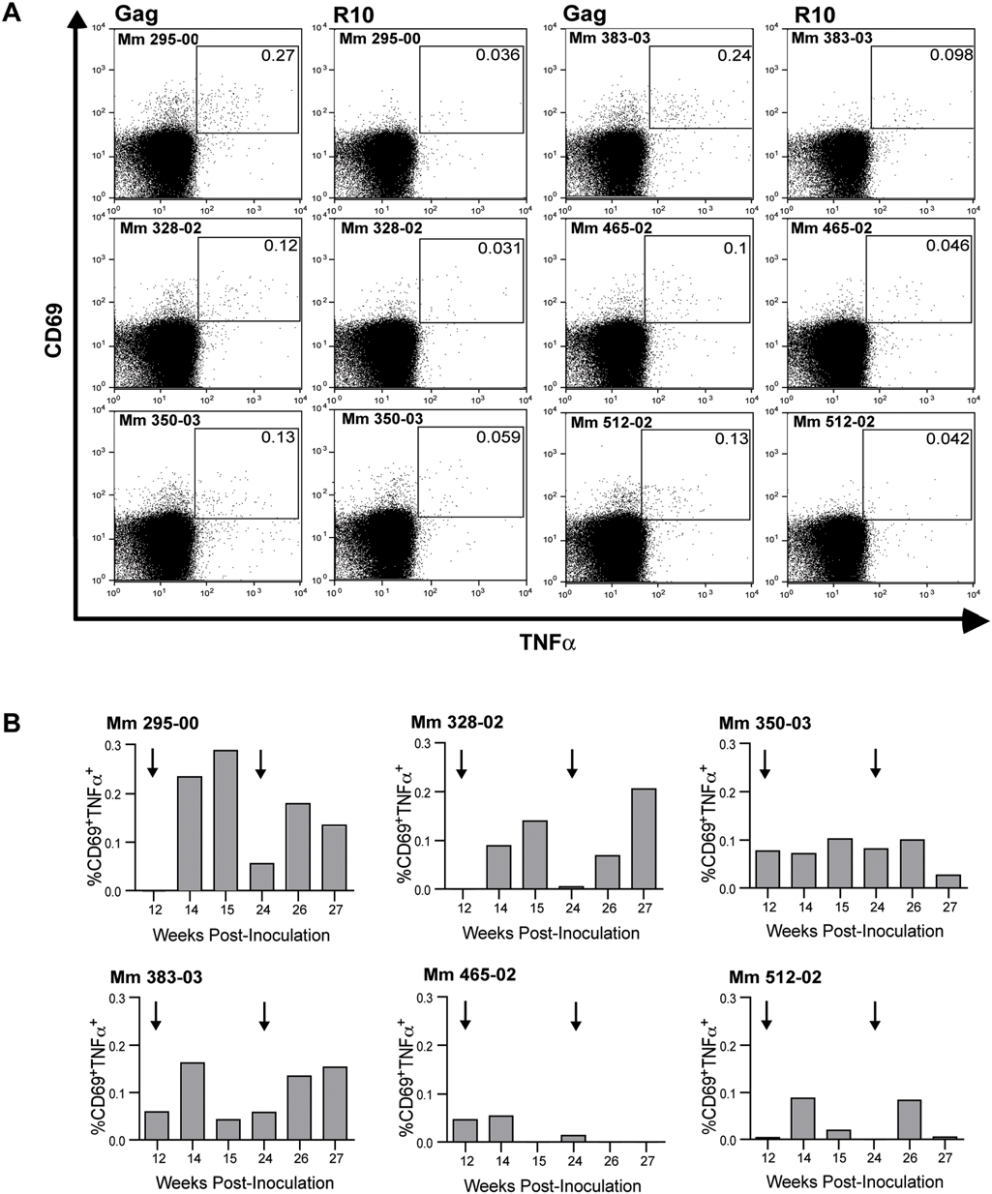
Immunization with single-cycle SIV elicits virus-specific CD4^+^ T helper cell responses. SIV Gag-specific CD4^+^ T cell responses were measured by intracellular cytokine staining. (A) CD4^+^ T cell responses are shown for each of the animals in Group B two weeks after the first boost (week 14). The gated populations indicate CD4^+^ T cells that have upregulated CD69 and TNFα after a 6-hour incubation in medium alone (R10) or in medium containing a set of overlapping Gag peptides (Gag). (B) Changes in the frequency of Gag-specific CD4^+^ T cells after each boost are summarized as bar graphs. The percentages of activated CD69^+^TNFα^+^ cells reflect the difference between responses to the Gag peptide pool and background responses in R10 medium. The arrows indicate booster inoculations on weeks 12 and 24.

Virus-specific CD4^+^ T cell responses were also detectable after boosting with VSV G trans-complemented scSIV. PBMC were stimulated with a pool of overlapping Gag peptides in tubes coated with co-stimulatory antibodies to CD28 and CD49d, and CD4^+^ T cells expressing TNFα and CD69 were detected by intracellular cytokine staining according to methods described by Gauduin *et al.* [45]. Gag-specific CD4^+^ responses ranged from 0.10% to 0.27% (mean 0.17% CD4^+^ T cells) two weeks after the first boost (Figure 6A). Increased Gag-specific CD4^+^ T cell frequencies were also observed in four of the six animals after the second boost (Figure 6B). Virus-specific CD4^+^ T cell responses are often weak or undetectable in HIV-1 infected people and SIV-infected animals due to ongoing virus replication and turnover of CD4^+^ lymphocytes, [46],[47]. Thus, the detection of CD4^+^ T cell responses following immunization with strains of SIV that are limited to a single round of infection may reflect the uncoupling of CD4^+^ T cell activation from ongoing infection and destruction of these cells.

### Antibody responses elicited by scSIV neutralize lab-adapted, but not primary, isolates of SIV

**Figure 7:**
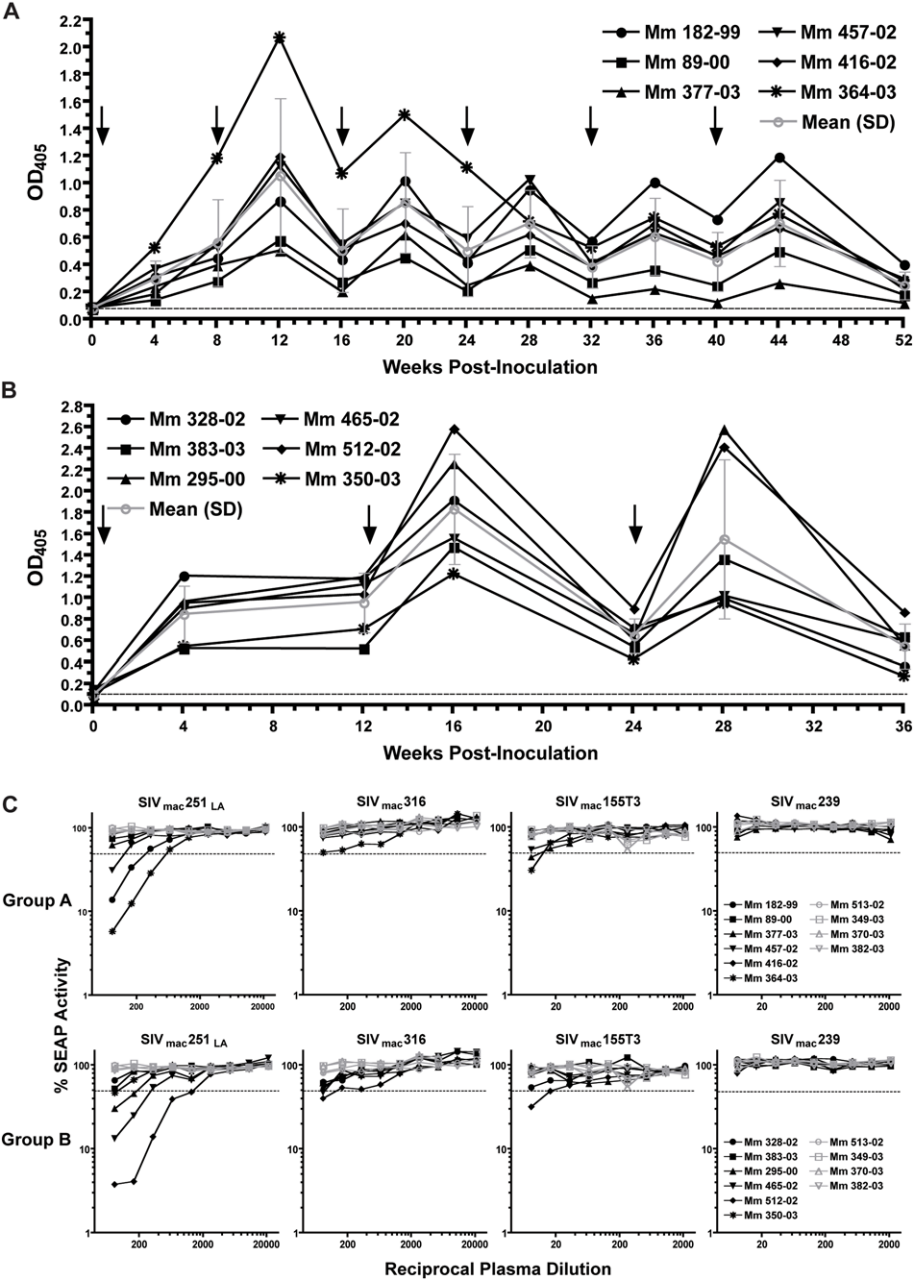
Both immunization regimens elicited antibody responses capable of neutralizing lab-adapted, but not primary isolates of SIV. Binding antibody responses were measured for Group A (A) and Group B (B) animals at a 1/20 dilution of plasma on ELISA plates coated with whole-virus lysate. The mean and standard deviation (error bars) of the antibody responses in each group are plotted in gray. Arrows indicate the time points of each inoculation, and the dashed line indicates the mean level of non-specific binding for pre-immune plasma at the time of the first inoculation. (C) SIV-specific neutralizing antibody titers at the time of challenge. Serial two-fold dilutions of plasma were tested for the ability to inhibit infection of target cells harboring a Tat-inducible SEAP reporter gene by lab-adapted SIV_mac_251 (SIV_mac_251_LA_), SIV_mac_316, SIV_mac_155T3, and SIV_mac_239. The dashed line in each plot indicates 50% neutralization of virus infectivity.

Antibody responses to SIV were monitored longitudinally by whole-virus ELISA. Plasma samples were tested at the time of each inoculation and four weeks later for antibodies capable of binding to plates coated with a lysate of purified virus particles. SIV-specific antibody responses were detectable in all of the animals by four weeks after the first inoculation (Figure 7). In Group A, binding antibody responses waxed and waned with each inoculation, but did not show an overall increase in titer (Figure 7A). In Group B, significant increases in virus-specific antibody titers were observed after the first boost at week 12 (P<0.001) followed by similar responses after the second boost at week 24 (P = 0.04) (Figure 7B).

At the time of challenge, neutralizing antibody titers were measured against four different strains of SIV; a neutralization-sensitive, lab-adapted strain of SIV_mac_251 (SIV_mac_251_LA_), and three primary isolates, SIV_mac_316, SIV_mac_155T3 and SIV_mac_239, matched in envelope with the scSIV strains included in the inoculum. Plasma samples from three of the animals in Group A and four of the animals in Group B neutralized SIV_mac_251_LA_ (<50% SEAP activity) at titers >80 (Figure 7C). Thus, both immunization regimens elicited antibodies capable of binding to the native, oligomeric conformation of envelope as it exists on virions. However, little or no neutralization was observed for the three primary isolates. While plasma samples from a couple of the animals in each group had measurable neutralizing activity against SIV_mac_316 and SIV_mac_155T3 at the lowest dilutions tested, none of the animals made detectable neutralizing antibody responses against SIV_mac_239 (Figure 7C). The inability to detect neutralizing antibodies to SIV_mac_239 is not particularly surprising given the inherent resistance of the 239 envelope glycoprotein to antibody-mediated neutralization, even with plasma from animals persistently infected with this virus [44],[48].

### Serotype-specific neutralizing antibodies to VSV G are elicited by VSV G trans-complemented virus that can be circumvented by changing VSV G serotypes

**Figure 8:**
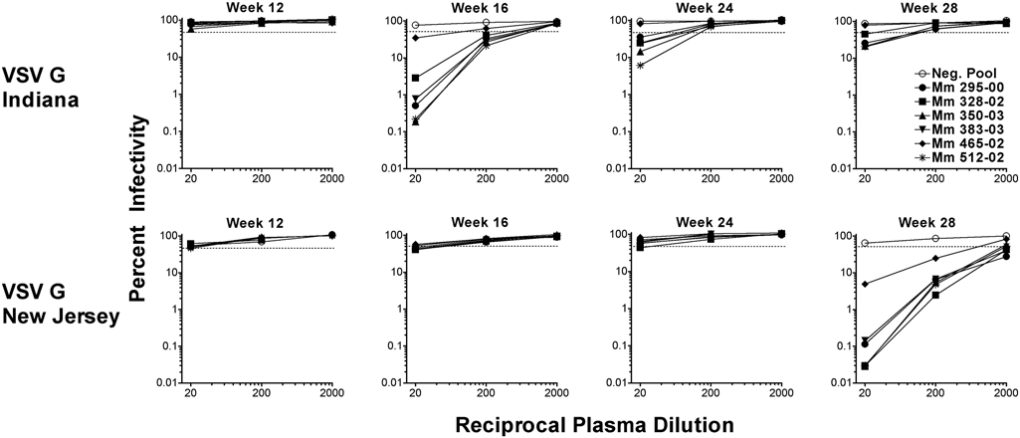
Neutralizing antibody responses to VSV G are serotype-specific. Plasma samples from Group B animals were tested for the ability to inhibit infection of CEM×174 cells by a VSV G-pseudotyped, *env*-deficient strain of SIV that expresses EGFP from the *nef*-locus. Neutralization curves for SIVΔenvEGFP pseudotyped with the Indiana and the New Jersey serotypes of VSV G are shown in the top and bottom rows, respectively. The percentage of infected, EGFP^+^ cells for virus incubated with plasma relative to virus incubated without plasma was determined by flow cytometry after 4 days of infection. Pooled plasma from three naive donor macaques was used as a negative control (Neg. Pool). The dotted line indicates 50% neutralization of virus infectivity.

Plasma samples from Group B animals were also monitored for neutralizing antibody titers to VSV G. Ten-fold dilutions of plasma were tested for the ability to inhibit infection of CEM×174 cells by an *env*-deficient strain of SIV that was pseudotyped with either the Indiana or the New Jersey serotype of VSV G. While none of the animals had neutralizing antibody titers against either serotype at the time of the first boost (week 12), some non-specific inhibition of infectivity was observed at the lowest dilution of plasma tested (Figure 8). This effect was greater for virus pseudotyped with the New Jersey serotype than for virus pseudotyped with the Indiana serotype of VSV G, an observation that may account for the lower peak of viremia for VSV G_NJ_ scSIV than for VSV G_I_ scSIV (Figure 2B). Nevertheless, four weeks after boosting with VSV G_I_ scSIV (week 16), plasma samples from each of the six animals neutralized virus pseudotyped with the Indiana serotype of VSV G, but not the New Jersey serotype of VSV G (Figure 8). Conversely, four weeks after boosting with VSV G_NJ_ scSIV (week 28), neutralizing antibody titers to the New Jersey serotype of VSV G (50% neutralization titer >500) were detectable in plasma from all six animals at a time when neutralizing antibody titers to the Indiana serotype were waning (Figure 8). These results are consistent with previous studies demonstrating that the Indiana and the New Jersey serotypes of VSV G do not elicit cross-reactive neutralizing antibodies in animals infected with VSV glycoprotein exchange vectors and validate our decision to change VSV G serotypes for each boost [10],[39].

### Both immunization regimens afforded significant containment of SIV_mac_239 replication and reduced memory CD4^+^ T cell loss after challenge

**Figure 9:**
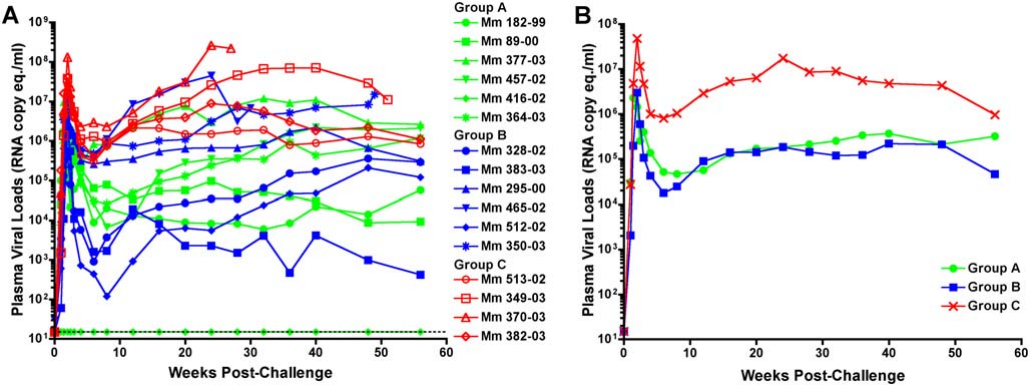
Both immunization regimens afford significant containment of SIV_mac_239 replication after challenge. Each of the scSIV-immunized animals in Groups A and B and four unimmunized control animals (Group C) were challenged intravenously with 10 animal infectious doses (1.5 pg p27) of SIV_mac_239. Viral loads for individual animals (A) and the geometric means for each group (B) are shown. Viral RNA loads were determined using a quantitative reverse-transcription-PCR assay with a threshold of detection of 15 copy eq./ml (dotted line). Mm 416-02 did not become infected and was not included in the calculation of the geometric mean for Group A. At the time that this manuscript was prepared, 7 of the 16 animals in this study had been euthanized with symptoms of AIDS. These animals included Mm 370-03 (week 27), Mm 465-02 (week 30), Mm 350-03 (week 49), Mm 349-03 (week 51), Mm 377-03 (week 60), Mm 295-00 (week 65), and Mm 364-03 (week 68). The clinical conditions of these animals at the time of euthanasia are described in the Materials and Methods section.

Twelve weeks after the last inoculation, each of the immunized animals in Groups A and B, and four unvaccinated control animals (Group C), were challenged intravenously with 10 animal infectious doses of SIV_mac_239. With the exception of a single animal in Group A (Mm 416-02), all of the animals became infected (Figure 9A). This animal may in fact have been fully protected, since the same stock of SIV_mac_239 has been used extensively by our group, and by others, without a single failure to establish infection in an unvaccinated control animal by the intravenous route of challenge [12],[28],[49]. However, we could not identify any differences in the immune responses of Mm 416-02 that might account for its resistance to infection. Furthermore, single-cycle viral RNA load measurements after each dose of scSIV were similar to the other animals in Group A suggesting that there was no inherent genetic barrier to infection of this animal. Therefore, to avoid inappropriately biasing our interpretation of the outcome of challenge, this animal was not included in the statistical analysis of post-challenge viral loads and CD4^+^ T cell counts.

Statistically significant reductions in viremia were observed in both immunized groups relative to the control group in both acute and chronic phases of infection. Area under the curve (AUC) analysis revealed significant containment of total viral loads for Group A (P = 0.03) and for Group B (P = 0.04) (Figure 9). Peak viral loads measured within the first 2 weeks post-challenge were 12-fold lower for Group A (P = 0.005) and 16-fold lower for Group B (P = 0.003), and set-point viral loads measured weeks 12–16 post-challenge were 52-fold lower for Group A (P = 0.009) and 33-fold lower for Group B (P = 0.05). In contrast to previous vaccine studies in which viral loads in immunized and control animals were indistinguishable by 40 weeks post-challenge [12],[13], these differences in chronic phase viral loads were stable for more than one year after infection (Figure 9). Indeed, comparisons of the geometric means of viral loads for each group over weeks 12–56 post-challenge using a linear mixed model analysis indicated that these chronic phase differences were statistically significant for both Group A (P = 0.015) and B (P = 0.014). Thus, both immunization regimens afforded significant containment of virus replication after an intravenous challenge with SIV_mac_239.

**Figure 10:**
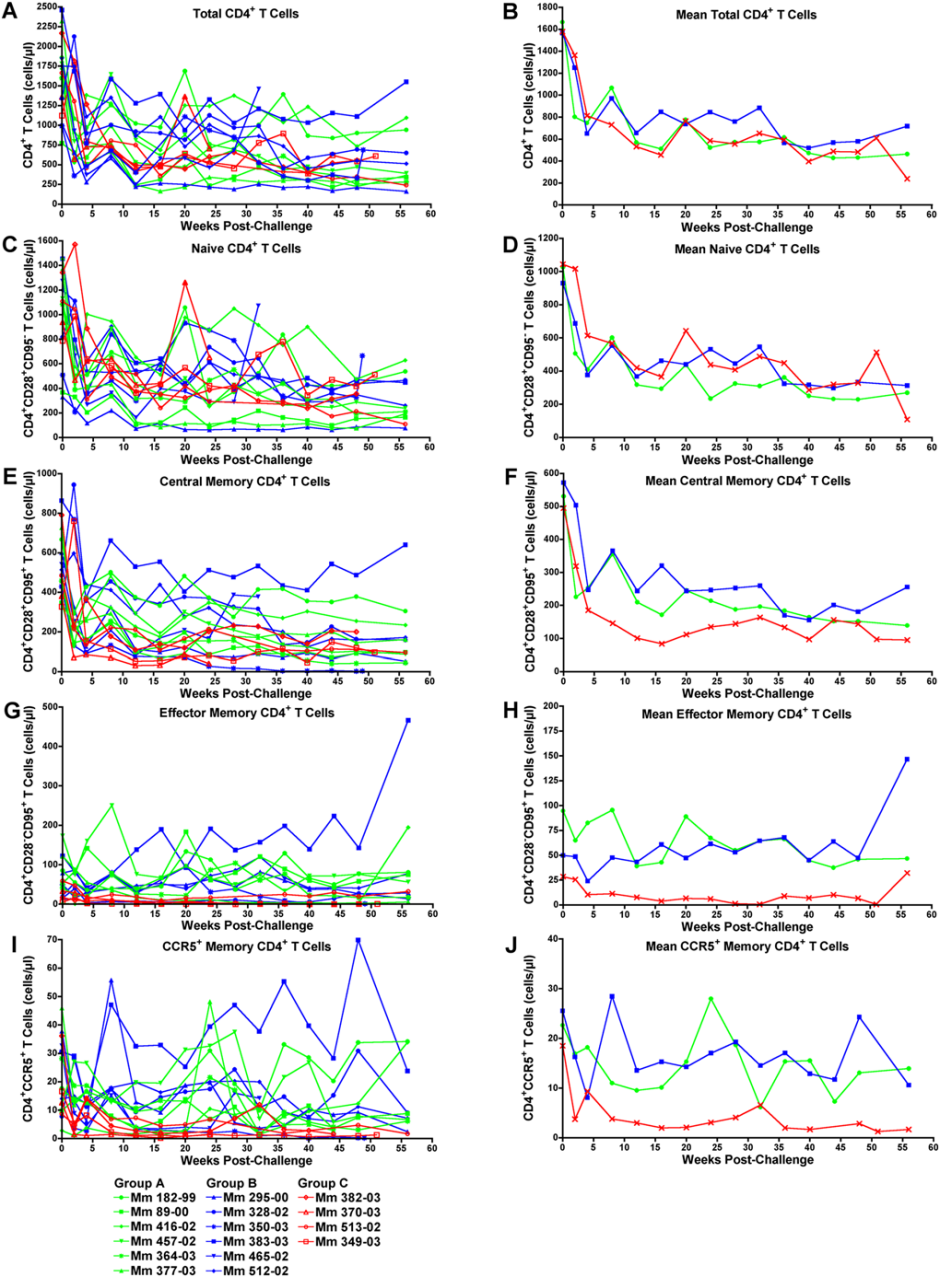
Memory CD4^+^ T cell subsets are better preserved in scSIV-immunized animals after challenge. Post-challenge CD4^+^ T cell counts in peripheral blood are shown for total (A,B), naive (C,D), central memory (E,F), effector memory (G,H), and CCR5^+^ memory (I,J) subsets. CD4^+^ T cell counts for individual animals are shown on the left (A,C,E,G,I), and the arithmetic means for each group are shown on the right (B,D,F,H,J). Whole blood was stained with monoclonal antibodies to CD3, CD4, CD28, CD95, and CCR5 to differentiate naive (CD28^+^CD95^−^), central memory (CD28^+^CD95^+^), effector memory (CD28^−^CD95^+^), and CCR5^+^ memory (CD95^+^) CD4^+^ T cells. The percentages for each population were determined by flow cytometry, and total cell counts were determined by complete blood count analysis at each time point.

Differences in the loss of memory CD4^+^ T cells were also observed for immunized versus control animals. There were no differences in the decline of total or naive CD4^+^ T cells (P = 0.7 and P = 0.2 respectively) (Figure 10A–10D). However, AUC analysis indicated significantly better preservation of the memory CD4^+^ T cell subsets in each of the immunized groups (Figure 10E–10J). The central, effector and CCR5^+^ memory CD4^+^ T cell populations were all significantly higher in Group A than in Group C (P = 0.03, P = 0.03 and P = 0.01 respectively). Likewise, the central and CCR5^+^ memory CD4^+^ T cell populations were significantly higher in Group B than in Group C (P = 0.05 and P = 0.03). Only the comparison of effector memory CD4^+^ T cell counts in Group B versus Group C fell short of statistical significance (P = 0.06). These results are consistent with the preferential infection and turnover of memory CD4^+^ T cells by SIV_mac_239 [33], and provide additional evidence of partial protection by each immunization regimen.

To identify potential correlates of protection, associations between the immune responses elicited by scSIV and post-challenge viral loads were also explored by linear regression analysis. IFNγ T cell responses to Gag and Env, at peak and at the time of challenge, were compared to peak and set-point viral loads after challenge. Neutralizing antibody titers to SIV_mac_251_LA_ and binding antibody responses to whole-virus at the time of challenge were also compared to peak and set-point viral loads after challenge. Additional associations were tested between peak single-cycle viremia for each strain of scSIV and peak viral loads during the acute phase of infection. None of these comparisons revealed significant correlations between parameters measured during the immunization phase of the study and viral loads post-challenge. Therefore, similar to previous studies with live, attenuated SIV [50], no correlations were observed between immune responses elicited by scSIV and the outcome of challenge.

## Discussion

More than 25 years after the emergence of the global HIV-1 pandemic, the development of a safe and effective AIDS vaccine remains an elusive scientific challenge. Although passive transfer studies have demonstrated that high concentrations of broadly neutralizing antibodies in plasma can provide sterilizing immunity to SHIV infection of macaques [51]–[54], it is presently unclear how to elicit such antibody responses by vaccination. Thus, efforts of the vaccine community have focused on T cell-based vaccines designed to reduce viral loads in immunized individuals who later become infected with the goal of reducing the incidence of HIV-1 transmission to new hosts. This strategy is supported by epidemiological data showing that the risk of heterosexual HIV-1 transmission is directly related to viral loads of the donor, and that individuals with plasma viral loads less than 1500 RNA copies/ml rarely transmit their infections to their partners [55]. Several recombinant vaccine candidates have been developed based on this premise that are designed to stimulate potent cellular immune responses [8]–[11],[16],[17]. However, the extent of protection achieved by these vaccines has so far been disappointing [12]–[19]. Live, attenuated strains of SIV afford more reliable protection in animals, often achieving sterilizing immunity against closely related challenge viruses [1]–[3]. However, due to the potential to regain a pathogenic phenotype during persistent replication *in vivo*, there are justifiable safety concerns with the use of live, attenuated HIV-1 as a vaccine approach in humans [4]–[6].

To uncouple the activation of virus-specific immune responses from persistent virus replication, we devised a genetic system for producing strains of SIV that are limited to a single round of infection [25],[28]. Single-cycle SIV retains many of the potentially advantageous properties of live, attenuated SIV, including the expression of 8 of the 9 viral antigens, the absence of any vector-derived gene products, and the expression of the native, oligomeric conformation of envelope on the surface of infected cells and virions. However, unlike attenuated vaccine strains, scSIV is not replication-competent and cannot revert to a pathogenic phenotype as a result of the accumulation of compensatory genetic changes during persistent replication *in vivo*.

Contrary to our initial hypothesis of “immunization to extinction,” repeated immunization with scSIV did not lead to the progressive maturation of virus-specific immune responses or successive decreases in single-cycle viremia. While SIV-specific T cell responses were detectable by MHC class I tetramer staining and IFNγ ELISPOT assays after the first and second doses, subsequent rounds of inoculation did not result in additional recall responses. These results are similar to previous observations [28], but are nevertheless puzzling given that viral RNA load measurements in plasma indicate a consistent take of infection after each inoculation. One possibility is that the T cell responses elicited by the first two doses were sufficient to resolve later rounds of scSIV infection with faster kinetics, thereby curtailing additional T cell activation and proliferation. Alternatively, repeated immunization may have had a tolerizing effect, perhaps by eliciting regulatory CD4^+^ T cell responses or inducing a state of clonal anergy. While the immunological basis for this phenomenon is presently unclear, the mechanism(s) responsible for permitting a certain level of scSIV infection without a detectable expansion of virus-specific T cell responses may ultimately account, at least in part, for the inability of natural immunity to resolve wild-type HIV-1 or SIV infection.

In contrast, boosting with VSV G trans-complemented scSIV dramatically increased virus-specific T cell frequencies. One week after the first boost, SIV-specific CD8^+^ T cell frequencies to immunodominant epitopes in Gag, Tat and Nef ranged from 0.55% to 3.7% of the CD8^+^ T cell population in peripheral blood. These responses were more than 10-fold higher than peak responses elicited after the initial priming dose, and are comparable to peak CD8^+^ T cell responses after boosting with recombinant poxviral or adenoviral vectors [8], [11]–[13]. These responses are also similar to Gag_181–189_-specific CD8^+^ T cell frequencies following the acute phase of SIV_mac_239 infection, which typically range from 0.5% to 10% of circulating CD8^+^ T cells [56]. Since the particle doses of VSV G trans-complemented and non-trans-complemented scSIV were similar for each inoculation (ranged from 10 to 15 µg p27), the greater magnitude of T cell responses after boosting with VSV G scSIV presumably reflects increased virus infectivity, and hence greater numbers of scSIV-infected antigen presenting cells after immunization. However, we cannot exclude the possibility that VSV G may also have facilitated scSIV entry into dendritic cells, or other professional antigen presenting cells, that potently activate T cell responses.

Gag-specific CD4^+^ T cell responses were also detectable by intracellular cytokine staining after boosting with VSV G trans-complemented scSIV. Although most HIV-1 infected patients and SIV infected animals make virus-specific CD4^+^ T cell responses, these responses are usually kept in check by ongoing virus infection [46], [57]–[59]. Higher frequencies of virus-specific CD4^+^ T cells have been observed in certain individuals who are better able to control HIV-1 infection and in animals persistently infected with live, attenuated strains of SIV [57],[59]. These observations suggest that virus-specific CD4^+^ T cells may be important for controlling HIV-1 and SIV infection, or alternatively, that better containment of virus replication may reduce CD4^+^ T cell turnover. By uncoupling CD4^+^ T cell activation from ongoing infection and destruction of these cells, scSIV may facilitate the development of virus-specific CD4^+^ T cell responses. Since CD4^+^ helper T cells play a central role in the maintenance of effective antibody and CTL responses to viral infections [60]–[65], these responses are likely to be an important component of an effective AIDS vaccine.

Both immunization regimens elicited similar neutralizing antibody titers to a lab-adapted strain of SIV_mac_251, verifying the induction of antibodies capable of binding to the native, oligomeric conformation of envelope as it exists on virions. However, only a couple of the animals in each group made low-titer neutralizing antibody responses to SIV_mac_316 and SIV_mac_155T3, and none of the animals had detectable neutralizing antibodies to SIV_mac_239. These observations reflect the greater resistance of primary isolates to neutralizing antibodies, particularly for SIV_mac_239 [44], and highlight the difficulty of eliciting even strain-specific neutralizing antibodies to viruses that express neutralization-resistant envelope glycoproteins typical of naturally transmitted HIV-1 field isolates. These results also suggest that envelope-specific antibodies were probably not a significant factor in protection against SIV_mac_239 at the time of challenge, although they do not preclude a role for anamnestic antibody responses in the control of virus replication after infection.

Following an intravenous challenge with SIV_mac_239, statistically significant reductions in viral loads and higher memory CD4^+^ T cell counts were observed for both groups of scSIV immunized animals. Relative to the unvaccinated control group, peak and set-point viral loads were 12- and 52-fold lower for the animals immunized by repeated inoculation (P = 0.005 and P = 0.009), and 16- and 33-fold lower for the animals immunized by the prime and boost regimen (P = 0.003 and P = 0.05). Furthermore, both groups of immunized animals maintained significantly better control of virus replication during the chronic phase of infection for more than one year after challenge. Based on prior associations of viral loads with HIV-1 transmission rates in humans, these reductions in set-point viral loads are in a range that might be expected to have a significant impact on heterosexual transmission [22],[55].

While encouraging, the extent of protection achieved by immunization with scSIV in these studies was not as good as typically afforded by infection with live, attenuated SIV [1]–[3]. This difference may have important implications to our understanding of the mechanisms of protection by live, attenuated vaccine strains. In contrast to scSIV immunization, which activates virus-specific immune responses that appear to decline into memory after the clearance of productively infected cells, attenuated strains of SIV continuously stimulate antibody and T cell responses as a consequence of persistent virus replication. Persistent antigenic simulation may have significant qualitative and quantitative effects on virus-specific immune responses. Animals infected with SIV_mac_239Δnef develop unusually high virus-specific CD4^+^ T cell frequencies with predominantly effector memory phenotypes [59], presumably reflecting ongoing antigenic stimulation. These responses, which can represent up to 4–10% of circulating CD4^+^ lymphocytes, are considerably higher than the CD4^+^ T cell responses elicited by scSIV immunization in this study, or indeed by wild-type SIV infection in previous comparisons [59]. By providing a constant source of infected antigen presenting cells, attenuated vaccine strains may also maintain CD8^+^ T cell responses in an activated state that allows for more rapid recognition and clearance of productively infected cells. In support of this, Rollman *et al.* observed that Gag-specific CD8^+^ T cells from macaques infected with an attenuated strain of SIV degranulated within 30 minutes after stimulation versus more than 3 hours for Gag-specific CD8^+^ T cells elicited by vaccination with recombinant poxviral vectors [66]. In the case of antibody responses, ongoing replication by live, attenuated viruses may drive the affinity maturation of envelope-specific neutralizing antibodies. Although passive transfer of serum from animals infected with attenuated viruses has not conferred protection [67], the time dependence of protective immunity [3],[68], and associated changes in the avidity and conformational dependence of envelope-specific antibodies [69], suggest that affinity maturation of neutralizing antibody responses may contribute to protective immunity. Thus, persistent virus replication and immune activation may be essential to achieve the degree of protection afforded by live, attenuated SIV.

SIV_mac_239 is a notoriously difficult strain to protect against by vaccination, particularly by the intravenous route of challenge. Prime and boost vaccine regimens using recombinant DNA and either poxviral or adenoviral vectors that were able to effectively contain virus replication after challenge with SHIV89.6P afforded little or no protection against SIV_mac_239 [8], [11]–[13]. In some cases, these vaccine approaches succeeded in reducing acute phase viral loads by approximately one log, but did not provide long-term control of SIV_mac_239 replication during the chronic phase of infection [12],[13]. Similar reductions in acute phase viral loads without long-term control of virus replication have also been observed when protection was assessed by challenging with uncloned, pathogenic SIV_mac_251 [16],[17]. In one study, better containment of SIV_mac_239 replication during the chronic phase of infection was achieved by vaccination of animals with a multivalent DNA-prime, rAd5-boost approach [14]. However, all of the animals in this study were Mamu-A*01 positive, and since Mamu-A*01 is associated with better control of SIV infection [70], MHC class I immunogenetics may also have contributed to the better outcome of challenge in these animals [14]. Other vaccine studies that have reported more effective control of SIV_mac_239 replication have typically used rhesus macaques of Chinese or Burmese origin [71],[72]. Since genetic evidence suggests that Chinese and Indian origin rhesus macaques represent distinct populations that separated approximately 162,000 years ago [73], and Chinese origin rhesus macaques have significantly lower viral loads and slower courses of disease progression after SIV_mac_239 or SIV_mac_251 infection than Indian origin rhesus macaques [74],[75], genetic differences between these two populations may account for the better vaccine protection observed in Chinese origin rhesus macaques. Thus, with the notable exception of live, attenuated SIV, the control of SIV_mac_239 replication achieved in this study by immunization with scSIV was at least as good, if not better than, other vaccine approaches that have been evaluated in this challenge model. A better understanding of the differences in immune responses elicited by immunization of macaques with single-cycle SIV versus live, attenuated SIV may provide important insights into the design of more effective vaccines for protection against HIV-1.

## Materials and Methods

### Animals

All of the animals used for these studies were Indian origin rhesus macaques *(Macaca mulatta)*. These animals were housed in a biosafety level 3 containment facility at the New England Primate Research Center (NEPRC) and were maintained in accordance with standards of the Association for Assessment and Accreditation of Laboratory Animal Care and the Harvard Medical School Animal Care and Use Committee. Animal experiments were approved by the Harvard Medical Area Standing Committee on Animals and conducted according to the principles described in the *Guide for the Care and Use of Laboratory Animals* [76]. All the animals selected for this study were negative for simian retrovirus type D, SIV, simian T lymphotrophic virus type 1 and simian herpesvirus B.

All of the animals in this study were typed for the rhesus macaque MHC class I alleles Mamu-A*01, -A*02, -A*08, -A*11, -B*01, -B*03, -B*04, -B*08, -B*17 and -B*29. The MHC class I alleles present in each animal are summarized in Table 1. Since Mamu-A*01 is associated with better control of SIV infection [70], the number of Mamu-A*01 positive animals assigned to each group was balanced to avoid a genetic bias in the outcome of challenge due to overrepresentation of this allele. Animals that were positive for both Mamu-A*01 and -B*17, or Mamu-A*01 and -B*08, and thus more likely to spontaneously control SIV infection independent of vaccination [70], [77]–[79], were excluded. MHC class I typing was performed by allele-specific PCR from genomic DNA using the primers and reaction conditions described by Kaizu et al. [80]. These assays were performed in Dr. David Watkins' laboratory at the Wisconsin National Primate Research Center (WNPRC, Madison, WI).

**Table 1:** MHC class I alleles present in the rhesus macaques selected for this study.

Group	Animal	A*01	A*02	A*08	A*11	B*01	B*03	B*04	B*08	B*17	B*29
A	Mm 182-99	+	−	−	−	−	−	−	−	−	−
	Mm 89-00	+	−	−	−	−	−	−	−	−	−
	Mm 377-03	−	+	−	−	−	−	−	−	−	−
	Mm 457-02	−	−	−	−	−	−	−	−	+	+
	Mm 416-02	−	−	−	−	−	−	−	−	−	−
	Mm 364-03	−	−	−	−	+	−	−	−	−	−
B	Mm 328-02	+	−	−	−	−	−	−	−	−	−
	Mm 383-03	+	+	−	−	+	−	−	−	−	−
	Mm 295-00	−	+	+	−	−	−	−	−	−	−
	Mm 465-02	−	−	−	−	+	−	−	−	−	−
	Mm 512-02	−	−	−	−	+	−	−	−	−	−
	Mm 350-03	−	−	−	−	+	−	−	−	−	−
C	Mm 513-02	+	−	−	−	−	−	−	−	−	−
	Mm 349-03	−	+	−	−	+	−	−	−	−	−
	Mm 370-03	−	−	−	−	+	−	−	−	−	−
	Mm 382-03	−	−	−	−	+	−	−	−	−	−

MHC class I typing was performed by allele-specific PCR as described in Kaizu *et al.* [80].

At the time that this manuscript was prepared, seven of the sixteen animals in this study had been euthanized with symptoms of AIDS. The week each animal died post-challenge, the experimental group the animal was assigned to, and the clinical conditions of the animal at the time of euthanasia are as follows. Mm 370-03 (Group C) was euthanized at week 27 post-challenge due to weight loss, persistent epistaxis and increased respiratory effort. Mm 465-02 (Group B) was euthanized at week 30 post-challenge with enlarged lymph nodes due to a progressive history of weight loss and diarrhea. Mm 350-03 (Group B) was euthanized at 49 weeks post-challenge due to periorbital edema, an enlarged spleen, an enlarged liver and agitated behavior. Mm 349-03 (Group C) was euthanized at 51 weeks post-challenge due to diarrhea and moderate weight loss with enlarged spleen and lymph nodes. Mm 377-03 (Group A) was euthanized at week 61 post-challenge with generalized lymphadenopathy and an enlarged spleen. Mm 295-00 (Group B) was euthanized at week 65 post-challenge with an enlarged spleen, lymphadenopathy and neurological symptoms. Mm 364-03 (Group A) was euthanized at week 68 post-challenge due to weight loss, diarrhea and lethargy.

### Sequence-tagged envelope variants of single-cycle SIV

Six different envelope variants of scSIV were constructed as previously described [32]. These included strains expressing full-length (TM_open_) and truncated (TM_stop_) forms of the 239, 316 and 155T3 envelope glycoproteins. The TM_stop_ strains contained a glutamic acid to stop-codon change at position 767 (E767*) that truncates the cytoplasmic domain of gp41 and results in increased envelope incorporation into virions and increased virus infectivity [35]. Unique 71–74 bp sequence tags selected from the *Arabidopsis thaliana* genome were introduced into the *pol* locus to allow independent quantification of viral RNA loads by real time RT-PCR analysis for each strain after mixed inoculation [32]. To facilitate the stimulation of virus-specific CTL responses, 26 residues from the C-terminus of the Nef protein that are required for MHC class I down regulation were eliminated by a glycine to stop-codon change at position 238 of Nef (G238*) followed by two single-nucleotide deletions (Δ9791 and Δ9797) [28],[37].

### Preparation of concentrated stocks of scSIV

Single-cycle SIV was produced by co-transfection of 293T cells with the Gag-Pol expression construct pGPfusion and a full-length proviral DNA construct for each scSIV strain as previously described [25],[28],[32]. 293T cells were seeded at 5×10^6^ cell per 100-mm dish in cell culture medium (Dulbecco's modified Eagle's medium [DMEM] supplemented with 10% fetal bovine serum [FBS], L-glutamine, penicillin and streptomycin) and transfected the following day with 5 µg of each plasmid using TransFectin™ Lipid Reagents (Bio-Rad, Hercules, CA). To produce VSV G trans-complemented scSIV, 5 µg of an expression construct for the Indiana or the New Jersey serotype of VSV G was included in the transfection mix. The cDNA clone for the New Jersey serotype of VSV G was kindly provided by Dr. John Rose (Yale University School of Medicine, New Haven, CT). Twenty-four hours after transfection, the plates were rinsed twice with serum-free medium and the cell culture medium was replaced with DMEM supplemented with 10% rhesus serum (Equitech-Bio, Kerrville, TX). Twenty-four hours later, the cell culture supernatant was collected and concentrated by repeated low speed centrifugation in YM-50 ultrafiltration units (Millipore, Bedford, MA). One-milliliter aliquots of scSIV were cryopreserved at −80°C and the concentration of virus was determined by SIV p27 antigen capture ELISA (Advanced BioScience Laboratories, Kensington, MD).

### Single-cycle infectivity assays

One million CEM×174 cells were incubated with 100 ng p27 equivalents of scSIV in 100 µl volume for 2 hours at 37°C. Cultures were then expanded to a volume of 2 ml in R10 medium (RPMI supplemented with 10% FBS, L-glutamine, penicillin and streptomycin) and incubated in 24-well plates at 37°C for 4 days. Cells were treated with Caltag Fix and Perm reagents (Caltag Laboratories, Burlingame, CA) and stained with the FITC-conjugated SIV Gag-specific monoclonal antibody 2F12 (provided by the DAIDS/NIAID Reagents Resource Support Program for AIDS Vaccine Development, under contract to Quality Biological, Inc. and Bio-Molecular Technology, Inc.; Principal Investigator, Ronald Brown; Project Officer, Jon Warren). After staining, cells were fixed in 2% paraformaldehyde PBS and analyzed by flow cytometry to determine the frequency of SIV Gag-positive infected cells.

### Immunization of macaques with scSIV

Six macaques (Group A) were inoculated intravenously with 6 identical doses of scSIV at 8-week intervals. Each dose contained a mixture of scSIV_mac_239 TM_open_, scSIV_mac_316 TM_open_ and scSIV_mac_155T3 TM_open_ (5 µg p27 eq. of each strain). Six additional animals (Group B) were inoculated intravenously with an initial priming dose that included scSIV_mac_239 TM_stop_, scSIV_mac_316 TM_stop_ and scSIV_mac_155T3 TM_stop_ (5 µg p27 eq. of each strain). These animals were then boosted on weeks 12 and 24 with VSV G trans-complemented scSIV_mac_239 TM_open_. The first boost contained 10 µg p27 eq. of scSIV trans-complemented with the Indiana serotype of VSV G (VSV G_I_) and the second boost contained 13 µg p27 eq. of scSIV trans-complemented with New Jersey serotype of VSV G (VSV G_NJ_). For each inoculation, 2–3 ml of concentrated scSIV was injected through a 22-gauge catheter placed aseptically in the saphenous vein of ketamine-HCI anesthetized animals (15 mg/kg intramuscularly).

### MHC class I tetramer staining

Virus specific CD8^+^ T cell responses were measured in the peripheral blood of Mamu-A*01 and -A*02 positive rhesus macaques. Whole blood (200 µl) was incubated for 30 min at 37°C with one of the following APC-conjugated tetramers provided by Dr. David Watkins' laboratory at the Wisconsin National Primate Research Center (University of Wisconsin, Madison, WI); Mamu-A*01-Gag_181–189_, Mamu-A*01-Tat_28–35_ and Mamu-A*02-Nef_159–167_. The samples were then stained with anti-CD3-FITC (clone SP34, BD Pharmingen) and anti-CD8-PerCP (clone SK1, BD Biosciences) monoclonal antibodies for an additional 30 min at room temperature. After staining, the samples were treated with FACS Lysing solution (BD Biosciences) to eliminate red blood cells, washed and fixed in 2% paraformaldehyde PBS. Data was collected using a FACSCalibur flow cytometer (BD Biosciences, San Jose, CA) and the frequency of CD8^+^ T cells staining with each tetramer was determined by analysis using the FlowJo software package (Tree Star, San Carlos, CA).

### IFN-γ ELISPOT assays

Virus-specific T cell responses were measured using enzyme-linked immunospot (ELISPOT) assay. PBMCs were plated at 3×10^5^, 1×10^5^ and 3×10^4^ cells per well in multiscreen 96-well plates (Millipore) coated with an IFNγ capture antibody (Mabtech, Mariemont, OH). PBMCs were stimulated in duplicate wells with peptide pools (15-mers overlapping by 11 amino acids, 2.5 µg/ml for each individual peptide) representing the amino acid sequences of the SIV Gag, Env, Nef, Tat, Rev, Vpr, Vpx and Vif antigens. Plates were incubated overnight at 37°C and developed using an enzyme-linked, colorimetric assay for bound IFNγ (Mabtech). Spots representing IFNγ-producing T cells were enumerated using an automated ELISPOT plate reader (Zellnet Consulting, New York, NY). The frequency of spot-forming cells (SFC) per million PBMC was calculated by subtracting the number of background spots in medium control wells from the number of spots in peptide-stimulated wells and adjusting for the input cell number.

### Intracellular cytokine staining

SIV-specific CD4^+^ T helper cell responses were detected by intracellular cytokine staining (ICS) as previously described [45]. Polystyrene flow tubes (12×75 mm) were coated overnight at a 5° angle at 4°C with 2.5 µg/ml goat anti-mouse IgG (H+L) (KPL, Gaithersburg, MD). The next day, the tubes were incubated with 10 µg/ml mouse anti-human-CD28 (clone CD28.2, BD Pharmingen) and mouse anti-human-CD49d (clone 9F10, BD Pharmingen) antibodies at 37°C for 1 hour. Fresh PBMC (1.5–2×10^6^) were stimulated at 37°C with the Gag peptide pool (2 µg/ml), SEB (100 ng/ml) or complete R10 medium in the presence of cross-linked co-stimulatory CD28 and CD49d antibodies. Brefeldin A (GolgiPlug, BD Pharmingen) was added after one hour and the incubation was continued for another five hours. After 6 hours of antigen stimulation, PBMC were surface-stained with anti-CD3-FITC (clone SP34, BD Pharmingen) and anti-CD4-PerCP monoclonal antibodies (clone L200, BD Pharmingen) at 4°C for 30 min. The cells were then treated with Fix & Perm reagents (Caltag Laboratories) and stained with anti-CD69-PE (clone FD50, BD Biosciences) and anti-TNFα-APC (clone Mab11, BD Pharmingen) at room temperature for 50 min. Cells were then fixed in fresh 1% paraformaldehyde PBS. Data were collected using a FACSCalibur flow cytometer collecting >200,000 lymphocyte events per sample and analyzed using the FlowJo software package.

### SIV-specific binding antibodies

SIV-specific binding antibodies were detected by whole-virus ELISA. Nunc-immunoplates (Fisher, Pittsburgh, PA) were coated overnight at room temperature with 0.1 µg p27 eq/ml whole-virus lysate prepared from aldrithiol-2 inactivated SIV CP-MAC (AIDS Vaccine Program, NCI-Frederick, Frederick, MD) [81]. Plates were blocked with a 1∶30 dilution of Kirkegaard and Perry BSA dilute/blocking solution concentrate (KPL, Gaithersburg, MD) and washed once with water. Duplicate 1∶20 dilutions of plasma were incubated in pre-treated wells for 1 hour. After 3 washes, 100 µl alkaline phosphatase conjugated Goat anti-human IgG (Fc) (KPL) at a dilution of 1∶100 was added to each well for 1 hour. The plates were then washed three times and 200 µl of phosphatase substrate solution (KPL) was added to each well. After 30 min, the reaction was stopped by the addition of 50 µl 3N sodium hydroxide and the absorbance was read at 405 nm.

### Neutralizing antibody responses to SIV

Neutralizing antibody titers were measured by the ability of plasma to block infection of target cells harboring a Tat-inducible secreted-alkaline phosphatase (SEAP) reporter gene [43]. Serial two-fold dilutions of plasma were incubated with lab-adapted SIV_mac_251 (0.25 ng p27 eq), SIV_mac_316 (5.0 ng p27 eq.), SIV_mac_155T3 (1.0 ng p27 eq.) or SIV_mac_239 (1.0 ng p27 eq.) in 96-well plates at 100 µl per well. After a one-hour incubaftion, 30,000 C8166 SIV-SEAP (SIV_mac_251_LA_, SIV_mac_239 and SIV_mac_155T3) or CEM×174-SEAP (SIV_mac_316) cells were added in an additional 100 µl R10 medium. SEAP activity was determined in culture supernatant collected on day 3 for SIV_mac_251_LA_, SIV_mac_239 and SIV_mac_155T3 and on day 5 for SIV_mac_316 using the Phospha-Light SEAP detection kit (Applied Biosystems, Foster City, CA). Mock-infected cells and SIV-infected cells incubated in the absence of plasma were used to determine background and maximal SEAP production respectively. After subtracting the background activity, percent neutralization was calculated by dividing the mean SEAP counts for replicate wells at each plasma dilution by the maximal SEAP counts in the absence of plasma.

### VSV G-specific neutralizing antibody responses

VSV G-specific neutralizing antibody responses were measured by testing 10-fold dilutions of plasma for the ability to inhibit infection of CEM×174 cells by a VSV G-pseudotyped, *env*-deficient strain of SIV that expresses EGFP from the *nef*-locus (SIV_mac_239ΔEnvEGFP) [82]. The expression of the SIV envelope glycoprotein by SIV_mac_239ΔEnvEGFP was disrupted by introducing a combination of nucleotide substitutions that changed the second and third codons of *env* to stop-codons and introduced a single nucleotide frameshift deletion (ATGGGATGTCTT -> ATGTGAT-AATT). VSV G-pseudotyped stocks of SIV_mac_239ΔEnvEGFP were produced by co-transfection of 293T cells with SIV_mac_239ΔEnvEGFP proviral DNA and expression constructs for either the Indiana or the New Jersey serotypes of VSV G. Ten-fold dilutions of plasma (1/20,1/200 and 1/2000) were incubated with 20 ng p27 eq. of VSV G-pseudotyped SIV_mac_239ΔEnvEGFP for one hour at 37°C in 100 µl R10 medium. CEM×174 cells (2.5×10^5^) were added, and the incubation was continued for an additional 2 hours in a total volume of 200 µl R10 medium. The cultures were expanded to one ml and transferred to 48-well plates. Four days later, the cells were harvested, fixed in 2% paraformaldehyde PBS and the frequency of infected EGFP^+^ cells was determined by flow cytometry. Percent infectivity was calculated by dividing the mean percentage of EGFP^+^ cells at each dilution of plasma by the mean percentage of EGFP^+^ cells in the absence of plasma and multiplying by 100.

### Intravenous challenge with SIV_mac_239

The immunized and control animals were challenged intravenously with SIV_mac_239. A vial of SIV_mac_239 challenge stock prepared in activated rhesus macaque PBMC (provided by Dr. Ronald Desrosiers, NEPRC) was thawed and diluted to 10 animal infectious doses (1.5 pg p27 eq.) per ml in serum-free RPMI 30 minutes prior to challenge. Under ketamine-HCl anesthesia (15 mg/kg, i.m.), each animal received one ml of the virus dilution through a 22 g catheter placed aseptically in the saphenous vein.

### Plasma viral RNA load measurements

Virus was recovered from 0.5 to 1.5 ml plasma collected in sodium citrate anticoagulant by centrifugation and viral RNA was extracted and reverse-transcribed into cDNA as previously described [83]. Single-cycle viral loads were measured using a quantitative, multiplex, real-time RT-PCR assay specific for unique sequence tags (*ggr*, *cao* and *gsa*) carried by each strain of scSIV. The primer/probe sets and reaction conditions for this multiplex assay are described in DeGottardi *et al.* [32]. Post-challenge viral RNA loads for SIV_mac_239 were measured using a standard quantitative, real-time, RT-PCR assay based on amplification of sequences in *gag* [83]. The nominal threshold of detection for this assay is 25 RNA copy eq. per ml and the interassay coefficient of variation is <25%.

### CD4^+^ T cell subsets post-challenge

The loss of total, naive and memory CD4^+^ T cell subsets was monitored after challenge. Whole blood was stained with the monoclonal antibodies CD3-FITC (clone SP34, BD Pharmingen), CD4-PerCP (clone L200, BD Pharmingen), CD95-APC (clone DX2, BD Pharmingen), and CD28-PE (clone CD28.2, BD Pharmingen) or CCR5-PE (clone 3A9, BD Pharmingen). Erythrocytes were eliminated by treatment with FACS Lysing solution (BD Biosciences) and the cells were fixed in 2% paraformaldehyde PBS solution. At each time point, the total number of lymphocytes was determined by complete blood count (CBC) analysis. Cell counts per µl of blood for each CD4^+^ T cell subset were calculated by multiplying the number of lymphocytes at each time point by the percentage of total, naive (CD28^+^CD95^−^), central memory (CD28^+^CD95^+^), effector memory (CD28^−^CD95^+^) and CCR5^+^ memory (CCR5^+^CD95^+^) CD4^+^ T cells.

### Statistical methods

Paired t-tests were used to assess differences in viral RNA load measurements and virus-specific T cell responses over time in the same animals. Independent sample t-tests were used to assess differences in these variables across groups at specific time points and to test differences between groups for area under the curve comparisons. Associations between immune responses elicited by scSIV and log-transformed, post-challenge viral RNA loads in plasma at peak and at set-point (week 12) were examined by linear regression analysis. Linear mixed models were also applied to analyze differences between groups in mean viral RNA load measurements during the chronic phase of infection (weeks 12–56 post-challenge) [84]. This method makes efficient use of all data points available and accounts for correlations between repeated measurements on the same animals. In this analysis, each animal was assumed to be completely randomized to one of three experimental groups, and thus assumed to be independent from each other. Animal-specific intercepts and group-specific slopes over time were included in the models. Viral loads and immunological variables were transformed using 10-based logarithmic whenever appropriate. Analyses were carried out using SPSS 15.0 software (SPSS Inc. Chicago, IL) and Strata MP 10.0 (Strata Corp., College Station, TX).
